# Aromatic Schiedam Schnapps

**Published:** 1853-01

**Authors:** 


*Aromatic Schiedam Schnapps.*—We have noticed in some of our contemporaries warm commendations of an article bearing the above prepossessing title, which, as we understand it, is but another name for *pure Holland gin.* The commendations to which we refer, appear to be redolent of said *schnapps*, and we doubt not that, after enjoying the same advantage for estimating the merits of this new medicinal article, we shall be equally *ardent* in its praise. We shall defer farther remarks till we have an opportunity of becoming more fully imbued with the *spirit* of the subject.

